# Packpred: Predicting the Functional Effect of Missense Mutations

**DOI:** 10.3389/fmolb.2021.646288

**Published:** 2021-08-20

**Authors:** Kuan Pern Tan, Tejashree Rajaram Kanitkar, Chee Keong Kwoh, Mallur Srivatsan Madhusudhan

**Affiliations:** ^1^ Bioinformatics Institute, Singapore, Singapore; ^2^ School of Computer Engineering, Nanyang Technological University, Singapore, Singapore; ^3^ Indian Institute of Science Education and Research, Pune, India

**Keywords:** missense mutation effect prediction, amino acid depth, local environment/clique, statistical potential, meta predictor

## Abstract

Predicting the functional consequences of single point mutations has relevance to protein function annotation and to clinical analysis/diagnosis. We developed and tested Packpred that makes use of a multi-body clique statistical potential in combination with a depth-dependent amino acid substitution matrix (FADHM) and positional Shannon entropy to predict the functional consequences of point mutations in proteins. Parameters were trained over a saturation mutagenesis data set of T4-lysozyme (1,966 mutations). The method was tested over another saturation mutagenesis data set (CcdB; 1,534 mutations) and the Missense3D data set (4,099 mutations). The performance of Packpred was compared against those of six other contemporary methods. With MCC values of 0.42, 0.47, and 0.36 on the training and testing data sets, respectively, Packpred outperforms all methods in all data sets, with the exception of marginally underperforming in comparison to FADHM in the CcdB data set. A meta server analysis was performed that chose best performing methods of wild-type amino acids and for wild-type mutant amino acid pairs. This led to an increase in the MCC value of 0.40 and 0.51 for the two meta predictors, respectively, on the Missense3D data set. We conjecture that it is possible to improve accuracy with better meta predictors as among the seven methods compared, at least one method or another is able to correctly predict ∼99% of the data.

## Introduction

Amino acid substitutions could affect protein stability, alter/impair its function, and possibly lead to disease conditions (Zhang et al., 2012). Several such single amino acid substitutions in proteins, also called missense mutations, are implicated in diseases such as cystic fibrosis, diabetes, cancer etc. (Roach et al., 2010; Stranger et al., 2011). Data from clinical studies and from large-scale projects such as the Human Genome Project (Craig Venter et al., 2001), the HapMap Project (Frazer et al., 2007), the Exome Sequencing Project, and the 1,000 Genomes Project (Altshuler et al., 2012) have unearthed such single amino acid mutations. It would be instrumental to have a fast and automated computational method to accurately predict the functional effect of these mutations. Such an exercise could also provide valuable insights into the development of personalized medicine.

Several computational methods predict the effect of missense mutations. The methods use sequence or structure information or a combination of the two. The sequence-based methods rely on previously known protein sequences and their characterizations deposited in databases. For example, in the SIFT method (Ng and Henikoff, 2003), mutational effect prediction is made based on a customized position-specific substitution matrix (PSSM), constructed using PSI-BLAST (Altschul et al., 1997) and MOTIF finder (Smith et al., 1990) to identify conserved local sequence regions. A majority of structure-based methods are based on machine learning algorithms. These methods employ different feature sets and machine learning architectures. For example, I-mutant2.0 (Capriotti et al., 2005) is trained on features such as pH, temperature, and mutation type using a support vector machine. AUTO-MUTE 2.0 (Masso and Vaisman, 2014) constructs a statistical contact potential with Delaunay tessellation and trains their models with additional attributes such as ordered identities of amino acids, pH, and temperature. PoPMuSiC-2.0 (Dehouck et al., 2009) uses a linear combination of 26 different statistical energy functions in an artificial neural network architecture. mCSM (Pires et al., 2014b) utilizes a graph metric to summarize physicochemical interactions within a cut-off distance as pattern signatures and trains them using a Gaussian process regression model. SDM (Pandurangan et al., 2017), which does not rely on machine learning, constructs an environment-specific amino acid substitution matrix based on observed substitutions in evolutionary time. DUET (Pires et al., 2014a) is a meta-algorithm that consolidates the methods of mCSM and SDM (Worth et al., 2011). Missense3D (Ittisoponpisan et al., 2019) is another structure-based method that uses 17 structural properties to predict the effect of the mutation. Dynamut2.0 (Rodrigues et al., 2020) uses normal mode analysis and graph-based signatures. Polyphen (Adzhubei et al., 2010) is a hybrid method that combines sequence and structural features to predict the effect of a mutation. It uses an improved version of PSSM, information from the Pfam database, and structural features such as accessible surface area and volume of an amino acid to make a prediction. SuSPect (Yates et al., 2014) is another hybrid-based method that uses PSSMs and Pfam domain profiles (Finn et al., 2014). It also includes information from protein–protein interaction networks and searches in the database for known functional annotations of a mutated position. Despite these various efforts and algorithms, the functional fate of point mutations remains a challenging problem.

A missense mutation could lead to functional instability by either disrupting its structure or by affecting its interaction interface and/or active sites without necessarily impacting its structure. A mutational effect predictor should hence take into account the effect of mutation on both overall structural stability and its functional relevance. In this study, we describe Packpred, which addresses both these aspects. For structural features, Packpred uses an environment-dependent multi-body statistical potential and a depth-dependent substitution matrix, FADHM. We had previously established that FADHM scores are useful in predicting the effects of point mutations (Farheen et al., 2017). The multi-body statistical potential considers the observed/expected ratio of cliques of residues. The greater the value of the ratio, the more energetically stable is the packing of amino acids in the residue clique. We further categorized these residue cliques based on their residue depths. Residue depth (Chakravarty and Varadarajan, 1999; Tan et al., 2011, Tan et al., 2013) measures the degree of burial and hence the solvation effect on amino acids. Depth has been shown to correlate well with the structural stability and free energy change of cavity-creating mutations in globular proteins (Chakravarty and Varadarajan, 1999; Tan et al., 2011). Our depth-based statistical potential hence assesses the effect of mutation on local packing stability. To capture the functional relevance of amino acids, we used residue position Shannon entropy from a multiple sequence alignment of homologs of the query sequence. By this, we exploit evolutionary information to quantify the degree of observed variation at the position of mutation. Usually, the lesser the variation, the greater is the functional importance of the residue.

## Materials and Methods

### Data Sets

#### Statistical Potential Data Set

A set of 3,753 protein structures (Supplementary Table S1) obtained from the Protein Data Bank (PDB) (Berman et al., 2000) was used to construct the clique statistical potential. The structures in this set have a resolution of 2.5 Å or better, an R-free of 0.25 or better, and are nonredundant at 30% sequence identity. To account for atomic position fluctuations (protein dynamics) while considering amino acid cliques, 10 homology models were built using Modeller9.11 (Šali and Blundell, 1993) with the native protein serving as both target and template in a self-alignment. The “refine very slow” option was used to relax the molecular structures with the aim of maximizing atomic position flexibility. These homology models along with the native structure (i.e., 11 structures for each protein) were then used to build the statistical potential.

#### Saturation Mutagenesis Data Sets

Saturation mutagenesis data sets of two proteins, T4-lysozyme (Rennell et al., 1991) and controller of cell division or death B (CcdB) (Adkar et al., 2012), were used in this study. T4-lysozyme is a 164 amino acid residue protein with our reference structure being PDB: 2LZM, which was solved at a resolution of 1.7 Å (Weaver and Matthews, 1987). Each position except the first was mutated to 13 other amino acids (A, C, E, F, G, H, K, L, P, Q, R, S, and T). After excluding key catalytic site residues (D10, E11, R145, and R148P), the data set consists of 1,966 mutations. CcdB is a cytotoxin (an inhibitor of DNA gyrase) with 101 amino acids. Its native structure was solved at a resolution of 1.4 Å [PDB: 3VUB (Loris et al., 1999)]. Full saturation mutagenesis (mutating each position to all other 19 amino acids) was performed at all positions of the protein. After removal of active site residues (I24, I25, N95, F98, W99, G100, and I101), a final set of 1,534 mutations was obtained. In both saturation mutagenesis experiments, an assessment was made on the phenotypic effect for each mutation. For T4-lysozyme, the phenotypic effect was gauged based on the plaque-forming ability of the mutant. Subject to the same experimental condition, a mutant is assigned to one of the four levels of sensitivity if the size of the plaque is (1) similar to native control, (2) significantly smaller, (3) with hazy morphology or difficulty in discerning plaques, and (4) no plaque formation (Rennell et al., 1991). For CcdB, the mutational sensitivity score was quantitatively defined as the titer number at which the protein activity (in this case, inducing cell death) decreases by 5-fold or becomes more relative to its previous dilution. Values of mutational sensitivity range from 2 to 9 in CcdB, and we scaled the T4-lysozyme values to range from 2 to 5. For both data sets, a mutation is regarded as neutral if there is no perceptible phenotypic difference as compared to its native sequence (MS score = 2 in CcdB and T4-lysozyme) and is regarded as destabilizing otherwise.

#### Missense3D Data Set

The Missense3D data set consists of 4,099 mutations from 606 proteins extracted from Humsavar (Bateman et al., 2017), ClinVar (Landrum et al., 2014), and ExAC (Karczewski et al., 2017) (Ittisoponpisan et al., 2019). Humsavar lists all the annotated missense variants from humans reported in UniProt and SwissProtKB. ClinVar catalogs variations in humans and their associated phenotypes. ExAC is an exome aggregation consortium that describes the aggregation and analysis of human exomes. The analysis includes quantification of the pathogenecity of variants. The data set of 4,099 mutations consists of 1,965 disease-associated variants and 2,134 neutral variants (not associated with any known disease yet). Packpred parameters were trained on the T4-lysozyme data set and tested on the CcdB and Missesense3D data sets.

### Structural and Sequential Features

#### Residue Depth

Depth is defined as the distance of a protein atom to the nearest bulk water molecule (Chakravarty and Varadarajan, 1999). The quantity measures the degree of burial of the atom. Depth has been shown to be capable of concisely describing the protein environment, as substantiated by its utilities in protein design and function predictions (Tan et al., 2011, Tan et al., 2013; Farheen et al., 2017). Atom depth values were computed using default parameters. The depth of a residue clique is defined as the average depths of its constituent atoms.

#### Cliques of Amino Acid Residues

**FIGURE 1:**
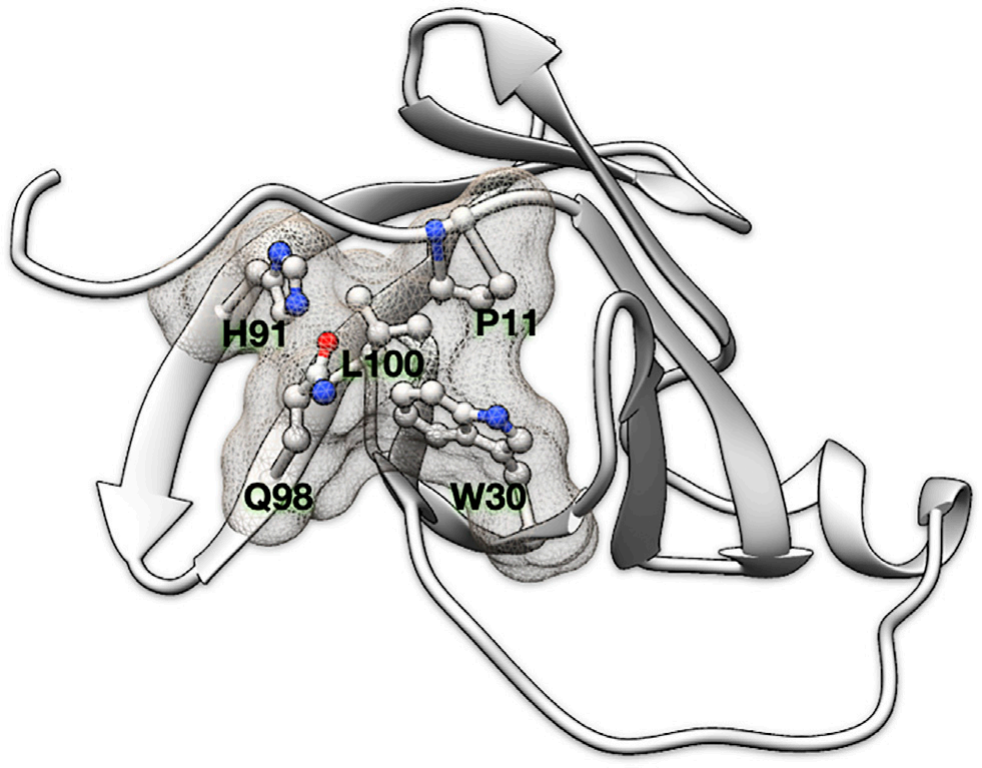
Residue clique of amino acids. A 5-residue clique (P11, W30, H91, Q98, and L100) of cut-off 7.5 Å shown in ball and stick representation and enveloped with a meshed molecular surface from human recombinant MTCP-1 protein (PDB: 1A1X).

A clique is defined as a sub-graph in which all possible pairs of vertices are linked. We define a (N, d_cut_) “residue clique” to be a clique of N amino acids within a linkage distance of d_cut_. We consider two amino acids as linked when at least four or more than half of the side chain non-hydrogen atoms (whichever are smaller) are within *d*_*cut*_ from atoms of another amino acid (Figure 1). For glycine, the C^α^ atom is used in lieu of the side chain. Residue cliques defined with different combinations of N and d_cut_ (N ranges from 2 to 4 and d_cut_ ranges from 7.0 to 10.5 Å in step of 0.5 Å) have been computed and investigated in this study.

#### Statistical Potential and Residue Clique Score

A residue clique statistical potential is constructed by adopting the formulation of Sippl’s potential of mean force (Sippl, 1990),

$$E^{c}=-kT\log\left(\frac{\left(P_{obs}^{c}+\alpha P_{\exp}^{c}\right)}{\left(P_{\exp}^{c}+\alpha P_{\exp}^{c}\right)}\right),$$ (1)

where *E* ^*c*^ is the pseudo potential energy and *c* is a residue clique of type {*r*_*1*_, *r*_*2*_, …}, where the *r*_*i*_’s are the amino acid types; P^c^_obs_ is the observed number of residue clique c; P^c^_exp_ is its expected number in a hypothetical reference state without energetic interactions; *α* is the ratio of pseudo-count introduced to account for sparse statistics and is taken as 0.00 in our study. −*kT* is a constant and is assumed as one in this study.

For each (*N*, *d*_*cut*_) clique, the statistical potential is built at five different levels of depth (2.80 – 5.25 Å, 4.25 – 6.25 Å, 5.25 – 7.25 Å, 6.25 – 8.25 Å, and 7.25 Å–∞). To calculate the score of a residue clique (S), the mean *μ* and standard deviation *σ* of its depth are first computed. A Gaussian probability density function *N*(*x* | *μ*, *σ*) is then accordingly built. The clique score is computed as the weighted sum of integrands at every depth level as follows:

$$S_{\mu ,\sigma }^{c}=\sum_{d\in D}\frac{1}{d_{f}-d_{i}}\overset{x=d_{f}}{\underset{x=d_{i}}{\int }}E_{d}^{c}\cdot N\left(x\vee \mu ,\sigma \right)dx,$$ (2)

where *d* is one depth level, and *d*_*i*_ and *d*_*f*_ are the lower and upper bounds of the level.

Most residue cliques in a protein are overlapping with one another, and an amino acid residue can participate in multiple cliques. The score of a residue is taken as the average of all such cliques (refer to Supplementary Text S1 for example). The score of a protein is further taken as the average of all its residue scores.

#### Shannon Entropy

Shannon entropy (H) is a measure of variation observed at a given position. It is calculated from a multiple sequence alignment obtained by a PSI-BLAST search against the uniref50 database (Altschul et al., 1997). H for a given position is then calculated as follows:

$$H=–\sum_{i=1}^{20}P_{i}\log_{2}P_{i},$$ (3)

where Pi is the fraction of amino acid i observed at a given position.

#### FADHM Scores

FADHM scores are depth-dependent pairwise amino acid substitution likelihood scores extracted from the FADHM matrices. The FADHM matrices quantify the substitution frequencies at different depths obtained by performing protein–protein structural alignments. A detailed account of the FADHM score can be found elsewhere (Farheen et al., 2017)

#### The Packpred Score for Mutations

The Packpred score is given as follows:

PS=1.5(S)+1.75(H)+0.5(FADHM), (4)

where PS is the Packpred score, S is the residue clique score obtained from the statistical potential, H is the Shannon entropy, and FADHM is the depth-based amino acid substitution likelihood score. The weights were obtained by training on the T4 saturation mutagenesis data set (Supplementary Table S2). The coefficients for S, H, and FADHM (weights) were systematically sampled in the range 0–3 with a step size of 0.25. The cut-off score threshold that best discriminates neutral mutations from destabilizing ones was 1.6 in the training data (see *Training and Testing Packpred Score*). Mutation with a score greater than 1.6 is neutral and is destabilizing otherwise. To score a mutant, we modify the clique composition without explicitly modeling the mutant protein structure, with the mutant amino acid inheriting all the properties of the wild-type residue.

Packpred is implemented as a web server at http://cospi.iiserpune.ac.in/packpred/. A standalone version is also available for download.

#### Matthews’s Correlation Coefficient

We gauge the binary classification performance of Packpred using Matthews’s correlation coefficient (MCC) (Matthews, 1975), which is given as follows:

$$MCC=\frac{TP\cdot TN–FP\cdot FN}{\sqrt{\left[TP+FP\right]\left[TP+FN\right]\left[TN+FP\right]\left[TN+FN\right]}},$$ (5)

where TP, TN, FP, and FN represent true-positive, true-negative, false-positive, and false-negative predictions.

## Results

### Training and Testing Packpred Score

Packpred uses a linear combination of sequence position Shannon entropy, a residue clique statistical potential, and a depth-dependent substitution matrix (FADHM) to predict the functional effect of missense mutations. The Shannon entropy part of the score estimates the functional importance of residues based on evolutionary information. The clique statistical potential and the substitution matrix gauge the effect of the mutation on the local environment/structure. The statistical potential computes the observed and expected probabilities to calculate a score for a clique. The FADHM scores are taken from substitution matrices that are derived from structural alignments of proteins. The substitution likelihood scores are calculated by categorizing a protein in three regions based on residue depths (exposed, intermediate, and buried). The substitution scores indicate the likelihood of a residue getting replaced by another at a given depth.

We performed a grid search in the range of 0–3 with a step size of 0.25 for S, H, and FADHM to optimize the coefficients (weights) of each component of the linear combination Packpred score. The optimization was to maximize Matthews’s correlation coefficient (MCC) (see below) of the T4 lysozyme saturation mutagenesis training data set. The weights that gave the highest MCC on the training set were 1.5, 1.75, and 0.5 for the clique statistical potential, Shannon entropy, and FADHM, respectively. We also obtained a cut-off threshold that distinguishes the destabilizing from the neutral ones from this training exercise. The cut-off was sampled in the range of 0–2 with a step size of 0.1. Mutations with scores greater than 1.6 are classified as neutral, and scores below 1.6 are classified as destabilizing. The T4-lysozyme training set consists of 1,362 (∼69%) neutral and 604 (31%) destabilizing mutations, of which Packpred correctly identifies 1,049 (∼77%) neutral mutations and 406 (∼67%) destabilizing mutations (Supplementary Table S3). In the T4 training exercise, we observe similar MCC values for different combinations of weights of the grid search. Although the MCCs are similar, the underlying predictions and the linear combination scores are different (refer to Supplementary Text S2 for an example).

The weights and threshold obtained from the training set were applied to two testing sets, the CcdB saturation mutagenesis data set (Supplementary Table S4) and the Missense3D data set (Supplementary Table S5). The CcdB data set has 1,258 (∼80%) neutral mutations and 276 (∼20%) destabilizing, while the Missense3D data set has 2,134 (∼52%) neutral and 1965 (∼48%) disease mutations, respectively. We used the PDB structures 2LZM and 3VUB to obtain Packpred scores of T4-lysozyme and CcdB, respectively. The biological unit of CcdB is a dimer, and we did all the calculations using this dimeric state structure for CcdB. Packpred correctly predicts 864/1,258 (∼68%) neutral and 253/276 (∼92%) destabilizing mutations from the CcdB testing set and 1,670/2,134 (∼78%) neutral and 1,123/1965 (∼57%) disease-causing mutations from the Missense3D data set.

We compared Packpred’s binary classification with several popular methods such as i-mutant2 (Capriotti et al., 2005), mCSM(Pires et al., 2014b), SDM(Pandurangan et al., 2017), dynamut2 (Rodrigues et al., 2020), FADHM(Farheen et al., 2017), and Missense3D (Ittisoponpisan et al., 2019) (Table 1). All the predictions were made using default parameters. Packpred was the best performing method on the T4-lysozyme training set and the Missense3D testing set, with MCC values of 0.42 and 0.36, respectively. The next best method is Missense3D with MCC values of 0.40 and 0.33 for the T4 and Missense3D data sets, respectively. The MCC of Packpred on the CcdB data set is 0.47 and is marginally outperformed by the best performing method, FADHM, which has an MCC of 0.48 (Table 1).

**TABLE 1:** Performance of some methods on T4, CcdB saturation mutagenesis, and Missense3D data sets.

Method	MCC for T4-lysozyme saturation mutagenesis data set	MCC for CcdB saturation mutagenesis data set	MCC for Missense3D data set
i-mutant 2.0	0.30^a^	0.36^a^	0.06
mCSM	0.22^a^	0.39^a^	0.05
SDM2	0.24^a^	0.33^a^	0.14
Dynamut2	0.09	0.15	0.06
Missense3D	0.40	0.39	0.33
FADHM	0.38^a^	**0.48** ^a^	0.27
Packpred	**0.42**	0.47	**0.36**

a Values taken from FADHM article.

The best MCC values are in bold.

The clique potential and FADHM were earlier trained on 3,753 and 2,384 PDB entries, respectively. 89 of these PDBs are common to the 606 PDB entries that comprise the Missense3D testing set (Supplementary Table S6). These 89 overlapping entries include not just those that are identical but also those that are homologs (with sequence identities of 30% or greater). The overlapping PDBs account for 463 of 4,099 mutations in the Missense3D data set. Omitting these 463 mutations and using the other 3,636 mutations resulted in an MCC of ∼0.37, comparable to the value of 0.36 obtained over the entire Missense3D data set of 4,099 mutations.

### Analysis of the Predictions on the Missense3D Data Set

The Missense3D data set has a balanced representation of ∼48% disease-associated mutations and ∼52% neutral mutations. The data set, however, is skewed in terms of amino acid abundance when compared to natural abundance (Supplementary Figure S1). For instance, arginine has the highest representation and accounts for ∼16% (664/4,099) of the Missense3D data set, while its natural abundance is ∼5%. The next most abundant amino acid in the Missense3D data set is glycine, which accounts for ∼9% (372/4,099) of the data (natural abundance is ∼7%). The most frequent mutant is also arginine (347/4,099), followed by serine (343/4,099). There are 2,233 mutations in the exposed environment (depth less than 5 Å), 1,258 in the intermediate environment (depth between 5 and 8 Å), and 608 in the buried environment (depth greater than 8 Å).

We assessed the performance of various methods on the Missense3D data set using metrics including sensitivity, specificity, precision, accuracy, and F1 (Table 2). Packpred outperforms all other methods in MCC, precision, and accuracy. Missense3D has the highest sensitivity and F1. Packpred has less sensitivity than FADHM and Missense3D, indicating potential for improvement. Packpred has a specificity of 0.57, indicating a higher number of false-positive predictions. mCSM and i-mutant outperform all other methods in specificity. However, mCSM, i-mutant, SDM, and dynamute predict a large number of false negatives (Table 3) that affect their MCC. Hence, we compare Packpred with FADHM and Missense3D in the next sections unless otherwise stated. Packpred has fewer false positives among FADHM and Missense3D and has the highest number of false negatives. The high false-positive rate contributes to its lower specificity.

**TABLE 2:** Prediction performance of seven methods on the Missense3D data set. The best score in each assessment metric is shown in bold font. Values of Class1 are used to describe the results in the manuscript.

Metric	Packpred	FADHM	Missense3D	Dynamut2.0	mCSM	i-mutant	SDM
MCC	**0.36**	0.27	0.33	0.06	0.05	0.06	0.14
Sensitivity (Class 0)	0.57	0.39	0.40	0.84	**0.92**	**0.92**	0.80
Specificity (Class 0)	0.78	0.85	**0.89**	0.20	0.10	0.12	0.34
Precision (Class 0)	0.71	0.71	**0.76**	0.49	0.49	0.49	0.52
F1 (Class 0)	0.63	0.50	0.53	0.62	**0.64**	**0.64**	0.63
Sensitivity (Class 1)	0.78	0.85	**0.89**	0.20	0.10	0.12	0.34
Specificity (Class 1)	0.57	0.39	0.40	0.84	**0.92**	**0.92**	0.80
Precision (Class 1)	**0.66**	0.60	0.62	0.59	0.59	0.60	0.63
F1 (Class 1)	0.72	0.70	**0.73**	0.31	0.18	0.20	0.44
Accuracy	**0.68**	0.62	0.65	0.51	0.50	0.50	0.55

**TABLE 3:** Confusion matrix values for the different prediction methods. The values in bold font show the best in each category. TP, FP, TN, and FN stand for true positive, false positive, true negative, and false negative, respectively.

Metric	Packpred	FADHM	Missense3D	Dynamut2.0	mCSM	i-mutant	SDM
TP	1,670	1816	**1890**	440	229	251	713
FP	842	1,203	1,177	312	**158**	164	420
TN	1,123	762	788	1,650	**1804**	1798	1,542
FN	464	318	**244**	1,685	1896	1874	1,412

We analyzed the results structurewise (Supplementary Table S7). Packpred correctly predicted all mutations from 264 (out of 606) structures and at least 50% mutations correctly from 507 structures. It could not correctly predict any mutation from 56 structures. In these 56 PDBs, the maximum mutations in any one protein were four, while the average number of mutations per PDB in the whole set was ∼6. These 56 structures did not follow any particular discernible pattern or trait.

Packpred has limitations in several areas. One of which is its high number of false-positive predictions that also affects its specificity. Other methods have a higher specificity but underperform in their sensitivity by overpredicting true negatives. Packpred has fewer true positives than FADHM and Missense3D, indicating another potential area for improvement. With more true positives, it is likely that Packpred’s F1 value would also improve, which is currently bested by Missense3D. Packpred has scores higher than 0.65 in all other metrics (accuracy, precision, sensitivity, and F1), indicating its overall balanced performance. We also calculated MCC (Supplementary Table S8) for each native amino acid type from the Missense3D data set. We found that of all 20 types of amino acids, Packpred has the highest MCC of 0.40 for Ile, Leu, and Val amino acids and the lowest MCC for Cys with an MCC of 0.17. Similar to Packpred, FADHM also has the lowest MCC of 0.04 for Cys amongst all the amino acid types. FADHM has the best MCC of 0.47 for I, which also happens to be the single best MCC for an amino acid among other methods. Missense3D, in contrast to Packpred and FADHM, has the best prediction for Cys with an MCC of 0.43 and has the lowest MCC of 0.02 for Trp among other amino acid types. These results show us amino acid–wise prediction performances and possibly contain useful hints on where one could improve the method.

**FIGURE 2:**
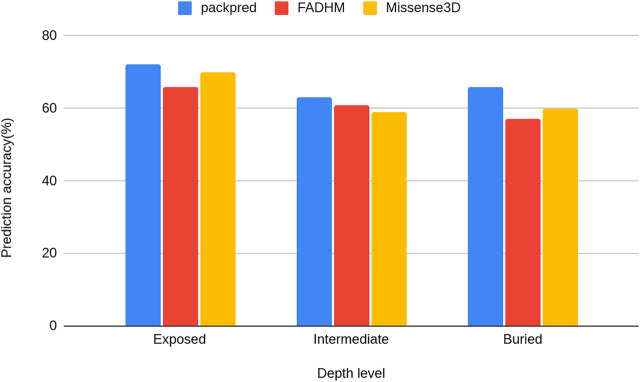
Histograms of the prediction accuracy of Packpred, FADHM, and Missense3D at different depth levels (exposed to the solvent, intermediate, and buried).

We stratified the Missense3D data to particular depth zones to assess the performance of these methods at particular depths. Packpred has 597/2,233 (∼72%) correct predictions from the exposed environment, 796/1,258 (∼63%) from the intermediate, and 400/608 (∼66%) from the buried environment. Packpred is the least accurate in predicting the effect of mutations in the intermediate environment. Interestingly, Missense3D is also the least accurate in this intermediate zone (Figure 2).

### Meta Predictions

Of the 4,099 mutants, at least one of the seven methods we tested made an accurate prediction in 4,036 cases. This motivated us to make two different meta predictions by combining the different methods.

The first meta prediction makes use of the method that performs the best for particular amino acids. We studied the wild-type (native) amino acid–wise trends of all seven methods. For instance, native amino acids N, K, Q, R, and T are best predicted by Missense3D, FADHM outperforms other methods in the prediction of I and M amino acids, and Packpred is the best at predicting A, D, E, G, L, P, V, and Y. In fact, all seven methods feature as the best method for at least one amino acid (Supplementary Table S9). Interestingly, we found that Packpred has the highest percentage (68%) of correct predictions when averaged over the 20 amino acids and with the lowest standard deviation (4%). In contrast, FADHM and Missense3D have averages of 62 and 64% with standard deviations of 7 and 10%, respectively. The other methods all have averages less than 60% with standard deviations between 11 and 14% (Supplementary Table S9). Packpred predictions are consistently well performing across the different native amino acid types. We then used these prediction strengths of each of the methods to get a hypothetical hybrid/meta prediction scheme (Supplementary Table S10) that combines predictions from all of the methods and has an MCC of 0.40 over the Missense3D data set, easily outperforming all the individual methods.

**FIGURE 3:**
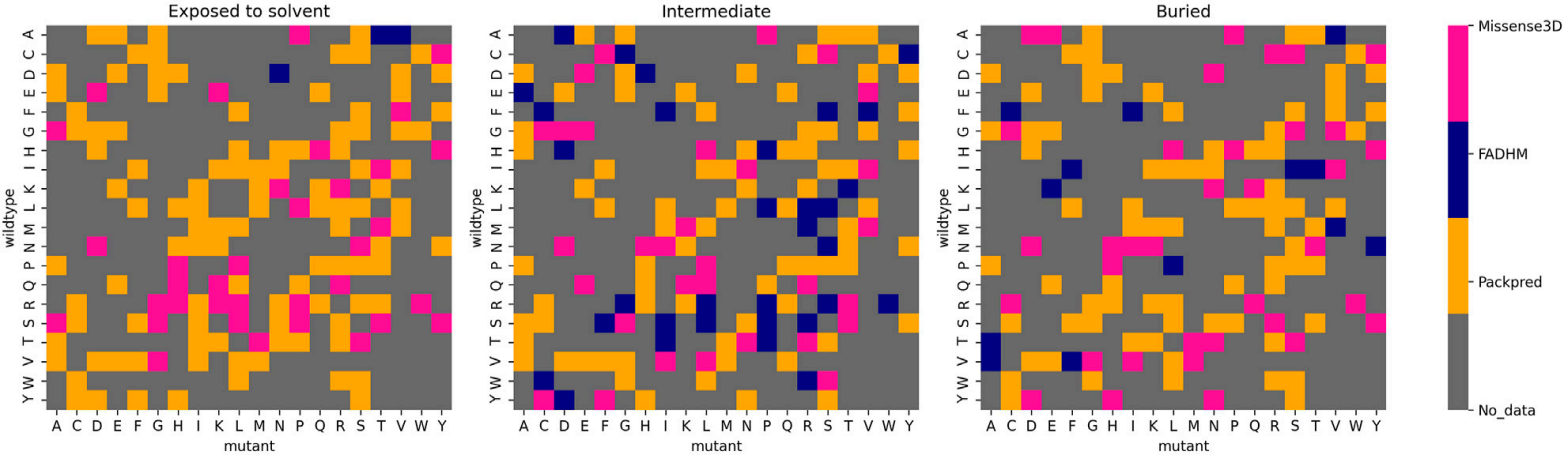
Best performing methods for each wild-type mutant amino acid pair at different depth levels.

The second hypothetical meta prediction only involves Packpred, FADHM, and Missense3D as these were the methods that did consistently well over all different data sets and amino acids. Here, we considered the method that best predicted wild-type mutant pairs. Furthermore, we segregated these amino acid pairs into different depth categories—exposed to the solvent (depth <5 Å), intermediate (depth between 5 and 8 Å), and buried (depth >8 Å). Our meta prediction then chose the best performing method for a particular pair at a particular depth level. For instance, the wild-type mutant pair A→D, Packpred has the best predictions in an exposed environment, FADHM in the intermediate environment, and Missense3D in the buried environment (Figure 3). In case of a tie between methods, the one with the better MCC was chosen. By thus combining the strengths of the three methods, the MCC of the predictions rises to 0.51 for the Missense3D data set (Supplementary Table S11). An analysis to rationalize/explain why certain methods are best for certain pairs/environments did not yield any illuminating results. It is clear, however, that there is some degree of complementarity in these different methods, and perhaps a more rigorous treatment of the results from the individual methods could further improve prediction accuracy.

We would like to emphasize here that the purpose of exploring these meta predictions was to simply test the extent to which we could possibly improve results with such an approach. In a more rigorous implementation of this method, we would have to train and test the meta-predictor separately, something that is beyond the scope of this study. Choosing the best results from our testing set, as we have done here, merely represents the possible limit up to which we could improve on predictions.

### Rank Ordering the Degree of Phenotypic Change by Mutations

We wanted to investigate if the Packpred scores are indicative of the degree of change/disruption caused by a mutation. The degree of change is measured experimentally using the mutational sensitivity score, which categorizes each mutation into one of four and eight levels in T4-lysozyme and CcdB data sets, respectively. We chose to use Spearman’s rank correlation coefficient (SCC) to measure the performance of rank-ordering, as it makes no assumption on a linear relationship between the scores and the phenotypical change. SCC is calculated as follows:

$$\rho =1–\frac{6\sum d_{i}^{2}}{n\left(n^{2}–1\right)},$$ (6)

where *d* is the difference between the actual and the predicted ranks of a mutation, and *n* is the number of levels. The SCC for T4 and CcdB data sets is −0.48 and −0.54, respectively. At best, this correlation is weak and indicates that these scores could be further improved.

### Assessing Robustness of Packpred

Last, we assessed the robustness of Packpred. For this, we changed the training set to include only 149 point mutations that result from a single nucleotide change in codons. The Missense3D data set is made of only these 149 different mutations. We created three additional training sets that all contain instances of only these 149 mutations. The first contains mutations from only the T4 lysozyme data set, the second set contains mutations from T4 in a 50:50 ratio of neutral-to-deleterious mutations, and the third set has mutations from the T4 and CcdB data sets in a 50:50 ratio of neutral-to-deleterious (Supplementary Table S12). The ratio was chosen based on the neutral-to-deleterious ratio of the Missense3D test set. For every combination of the training set, we obtained different optimal weights for the features of the linear combination (Supplementary Table S12). Interestingly, the accuracy of the method as gauged by the MCC value over the Missense 3D data set was consistently between 0.34 and 0.35 (Supplementary Tables S13–15).

## Discussions

In this study, we have developed a method to predict the effect of missense mutations on the structure and function of a protein. We believe that such predictions could be tested by assaying the protein for its function. Our method, Packpred, is constructed in a way that it is sensitive to structural changes effected by the mutation and any functional changes it may effect without perturbing the structures. To assess the impact of the mutation on the structure (and hence the function) of the protein, we devised a multi-body clique statistical potential. This statistical potential evaluates the strength of the interaction in a local neighborhood (amino acid clique). To assess the impact of mutation, we consider the same residue neighborhood environment while replacing the wild-type amino acid with the mutant. The score of the clique with the wild-type residue and with the mutant are then computed. An inferior score for the mutant in comparison to the wild type would be indicative of a destabilizing mutation. The structural stability of introducing the mutant residue is also gauged using a depth-dependent substitution matrix, FADHM, whose efficacy at detecting the fate of mutations we had previously benchmarked and tested. To account for functional changes that could happen even when the structure is not affected by the mutation, we invoke evolutionary information from a multiple sequence alignment using Shannon entropy. The more conserved the position, the more likely that it is going to affect function. These different scores are taken together in a linear combination, whose coefficients were optimized using the T4-lysozyme saturation mutagenesis data set of ∼2,000 mutations. Packpred was tested on two different data sets, another saturation mutagenesis data set (CcdB) and the Missense3D data set. Its performance on these data sets was also compared to those of six other methods including FADHM, Missense3D, Dynamut2.0, mCSM, i-mutant2.0, and SDM. With the exception of the CcdB data set, where it marginally underperforms FADHM, Packpred clearly outperformed all other methods on all data sets. Among the methods, Packpred balances well between predicting true positives and true negatives (neutral and disease-causing mutations) and hence has the best MCC values. Packpred has the best accuracy and is close to the best specificity, precision, and F1. It loses out to the best methods in these measures and on sensitivity as methods such as mCSM predict a disproportionately large number of negatives. When the performance of the different methods is compared on an (wild-type) amino acid by amino acid basis, Packpred performs consistently well, with prediction accuracies never falling below 60%, while maintaining an average of 68%, which is easily the best among the methods tested. Qualitatively, a similar picture also emerges when the results are broken down into wild-type mutant amino acid pairs.

We also investigated whether Packpred (and other methods) preferred certain types of structures over others. No clear deduction could be made from these analyses. However, there was one trend that could be considered for further improvements—Packpred, similar to Missense3D and FADHM, performed the worst in the intermediate amino acid depth environment. Mutational effects in exposed and buried (according to residue depth) environments were better predicted. Perhaps, the intermediate depth levels need to be further stratified, which in the case of Packpred would be reflected in the FADHM matrix values and in the clique statistical potential. Improvements could also be thought of by examining the reasons for why Packpred was unable to accurately predict the fate of 72 mutants that were all accurately called by the other six methods. We could also dissect the 23 correct predictions that Packpred made that were missed by all other methods to determine the relative strength of Packpred in comparison to the other methods.

Packpred relies on the sequence and structure of a given protein to predict the effect of a mutation. It is likely that these predictions could be impacted by the accuracy/resolution of the protein structure. The two structural features that Packpred extracts from structures are amino acid depth and structural neighbors. To whatever extent these two features get affected by the quality/accuracy/resolution of the structure would predicate the impact it would have on the final predictions. For the structures in the Missense3D data set, they all have resolutions of 2 Å or better. For this set, there appears to be no correlation between the accuracy of the prediction and the resolution of the structure (Supplementary Figure S2). In an independent study, we are exploring the use of homology models along with low-resolution structures from the PDB to quantify the impact of structural accuracy on Packpred predictions.

The clique statistical potential that has many tunable parameters such as the number of amino acids in the clique, cut-off distance, and definitions of what constitutes a “contact” between residues. Packpred could improve by investigating these aspects too, and this would form an independent study in itself. Similarly, further tweaks to the FADHM matrix, as briefly discussed above, could also possibly improve overall prediction accuracy. Shannon entropy accounts for the degree of variation at a given site/position and does not change depending on the type of mutation. In our method, we use Shannon entropy in conjunction with the clique potential and FADHM to get a wholesome picture of sequence and structure conservation. However, it is likely that a more nuanced version of the entropy measure and/or other scores for conservation may help get more accurate predictions. In its current implementation, Packpred categorizes mutations as being neutral or destabilizing. When we tried to correlate the score with a discretized value of the function, the correlations were around −0.5. Perhaps, with some of the improvements discussed above, this correlation would also improve.

One important observation from our findings is that of the 4,099 mutations, 4,036 were correctly called by at least one of the methods. There exists great complementarity between the methods tested here. We were tempted to then use two simple meta prediction methods. We designated the predictions involving a particular wild-type amino acid or a wild-type mutant amino acid pair to the method that best predicted this type. Such a simple-minded approach gave us MCCs of 0.40 and 0.51 for the amino acid and the amino acid pair type predictions, respectively, where the best predicting method, Packpred, had an MCC of 0.36 (Missense3D data set). It is conceivable that a different method of combining the results from these different methods could vastly increase the accuracy of predicting the functional fate of single amino acid changes.

We assessed the robustness of Packpred by training it on the T4 set and a combination of the T4 and CcdB saturation mutagenesis data sets. Each of the training sets gave us different optimal values of feature weights. These different weights did not, however, affect the overall performance of the method on the Missense3D testing set. In earlier results too, we had observed that different weight combinations gave rise to similar performances on the training set. We believe that one of the primary reasons for the different optimal weights is the fact that the three features in Packpred do not all affect predictions at the same level of granularity. The statistical potential and the substitution matrices (FADHM) give a score for particular mutations, whereas the Shannon entropy score gives a single value for a position, regardless of the type of mutation. Given the myriad of different environments and levels of conservation in different positions of the protein, the contribution due to each of these features is not uniformly the same across a protein. The positive aspect of these predictions is that despite the lack of consensus of optimal values of the different features, the overall prediction accuracy does not appear to suffer. This is probably indicative of the fact that the features of the algorithm are important, and perhaps a different way of combining these features may yield consistently better results.

## Data Availability

The original contributions presented in the study are included in the article/Supplementary Material; further inquiries can be directed to the corresponding author.
