# Supplementary material for: Packpred: Predicting the Functional Effect of Missense Mutations
Source: Front Mol Biosci. 2021 Aug 20;8:646288. doi: 10.3389/fmolb.2021.646288 (PMC8417552; doi:10.3389/fmolb.2021.646288)
Supplement: Supplementary file 2 [file DataSheet1.docx]

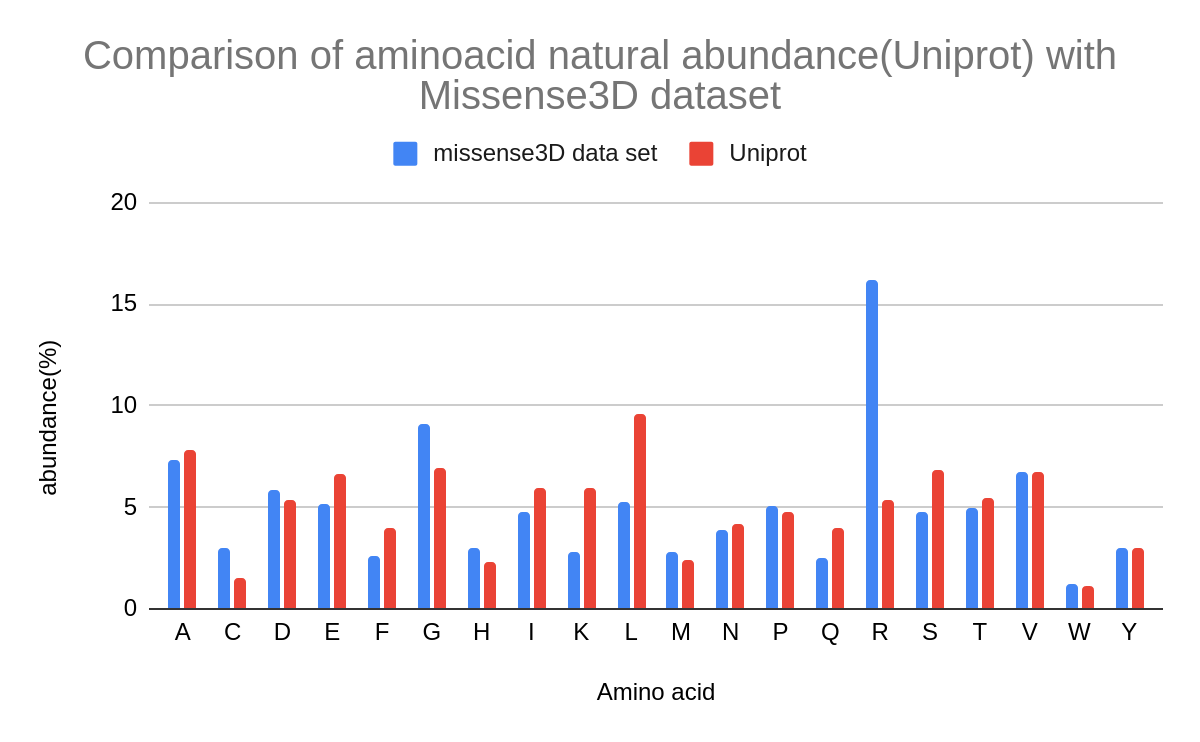


Supplementary Figure1: Abundance of amino-acids in Missense3D dataset (wildtype residues) with natural abundance (Taken from UniprotKB 8.0)


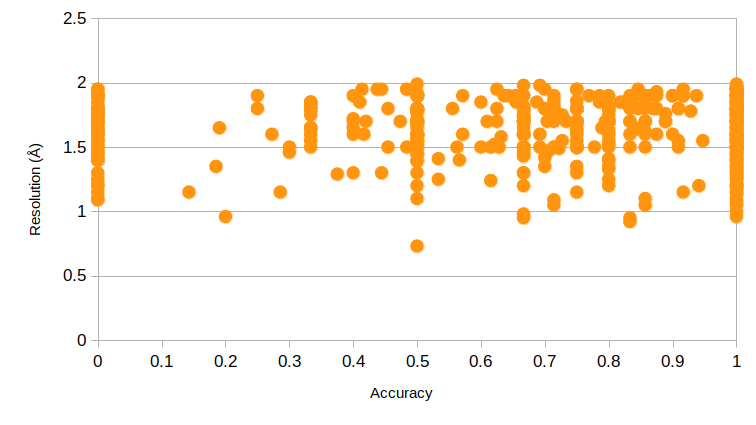


Supplementary Figure2: Correlation between PDB structure resolution(A) and the accuracy of predictions by Packpred

Supplementary text S1_text: Example of clique score calculation

Consider a four body clique consisting of L100, P11, Q98 and W30 at a distance cutoff of 7.5 from PDB 1A1X. The average residue depth of this clique is ~6.8 Å. The log(observed value) at this depth level of the LPQW clique is -9.83 and the log(expected value) is -9.82 leading to a log odd ratio of ~ -0.02. Negative scores are indicative of energetically favourable outcomes. The greater the negative score the more stable is the clique. This score is for the whole clique of 4 residues. For a residue-wise score, we should consider all cliques that the residue of interest has contributed to. (Supplementary Figure3). The log odd ratios of all the cliques that contain the residue are calculated (as demonstrated earlier). These scores are then used to calculate the score of a residue using equation (1).


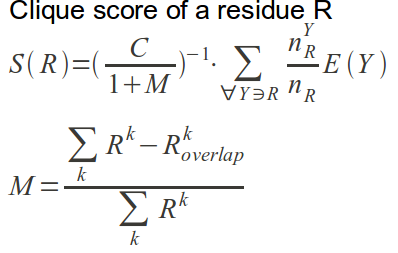


(1)

Where,

*Y* is the clique index, *C* is the total number of overlaps between cliques, *n_R_* is total number of atoms of residue *R*, *n^Y^_R_* is number of atom of *R* involved in clique *Y, k* is the atom index


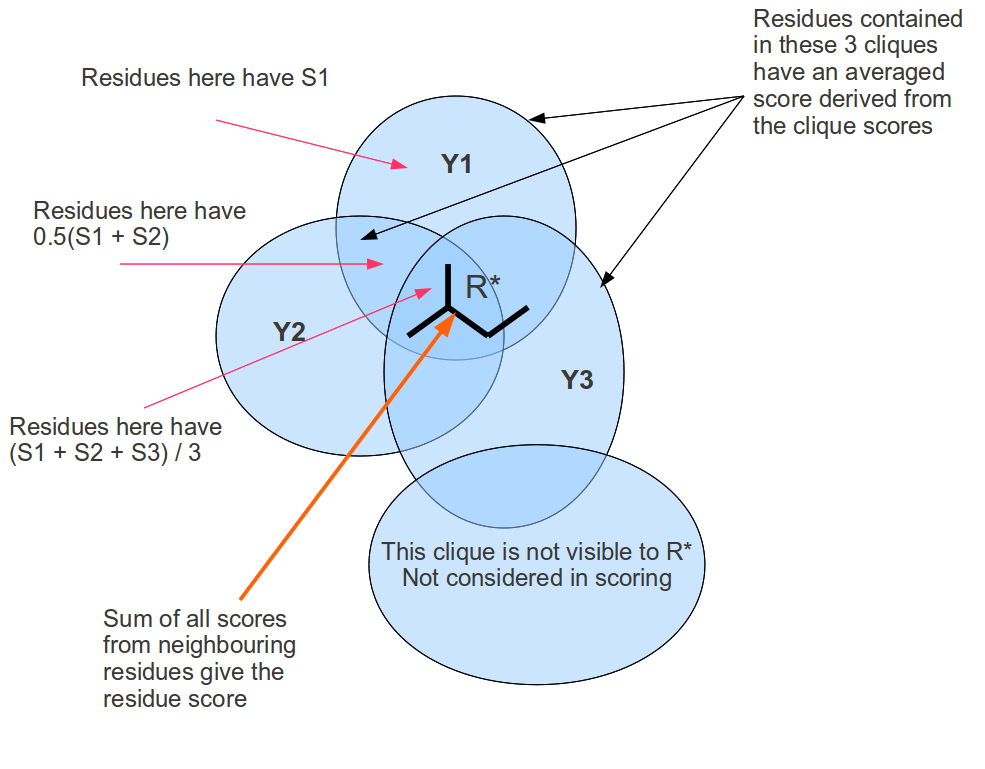


Supplementary Figure3: Illustration of clique residue score

Supplementary text S2_text: Example of similar MCC values from different parameter values

Consider these two sets of parameter values (best MCCs from the T4 lysozyme training) and their MCCs. Set1 = ( 1.5, 1.75, 0.75, cutoff of 1.6, MCC = 0.42) and Set 2 = (2.25, 3.00, 1.25, cutoff of 1.7, MCC = 0.42). There are 132 distinct and 1834 common predictions between these 2 sets. Although the 1834 mutations have identical predictions their raw scores are different.

Supplementary tables can be found in the accompanying Supplementary_data_tables.xls file
